# Possible Therapeutic Doses of Cannabinoid Type 1 Receptor Antagonist Reverses Key Alterations in Fragile X Syndrome Mouse Model

**DOI:** 10.3390/genes7090056

**Published:** 2016-08-31

**Authors:** Maria Gomis-González, Arnau Busquets-Garcia, Carlos Matute, Rafael Maldonado, Susana Mato, Andrés Ozaita

**Affiliations:** 1 Laboratory of Neuropharmacology-NeuroPhar, Department of Experimental and Health Sciences, Program of Genetics and Neurosciences, University Pompeu Fabra, Barcelona 08003, Spain; maria.gomis@upf.edu (M.G.-G.); arnau.busquets-garcia@inserm.fr (A.B.-G.); rafael.maldonado@upf.edu (R.M.); 2 Department of Neurosciences, University of the Basque Country UPV/EHU, Leioa 48940, Spain; carlos.matute@ehu.eus (C.M.); susana.mato@ehu.eus (S.M.); 3 Achucarro Basque Center for Neuroscience, Zamudio 48170, Spain; 4 Centro de Investigación Biomédica en Red de Enfermedades Neurodegenerativas (CIBERNED), Madrid 28031, Spain

**Keywords:** fragile X syndrome, synaptic plasticity, long-term depression, memory, treatment, cannabinoid receptor, CB1, endocannabinoid system

## Abstract

Fragile X syndrome (FXS) is the most common monogenetic cause of intellectual disability. The cognitive deficits in the mouse model for this disorder, the Fragile X Mental Retardation 1 (Fmr1) knockout (KO) mouse, have been restored by different pharmacological approaches, among those the blockade of cannabinoid type 1 (CB1) receptor. In this regard, our previous study showed that the CB1 receptor antagonist/inverse agonist rimonabant normalized a number of core features in the Fmr1 knockout mouse. Rimonabant was commercialized at high doses for its anti-obesity properties, and withdrawn from the market on the bases of mood-related adverse effects. In this study we show, by using electrophysiological approaches, that low dosages of rimonabant (0.1 mg/kg) manage to normalize metabotropic glutamate receptor dependent long-term depression (mGluR-LTD). In addition, low doses of rimonabant (from 0.01 mg/kg) equally normalized the cognitive deficit in the mouse model of FXS. These doses of rimonabant were from 30 to 300 times lower than those required to reduce body weight in rodents and to presumably produce adverse effects in humans. Furthermore, NESS0327, a CB1 receptor neutral antagonist, was also effective in preventing the novel object-recognition memory deficit in Fmr1 KO mice. These data further support targeting CB1 receptors as a relevant therapy for FXS.

## 1. Introduction

Fragile X syndrome (FXS), the most common form of inherited intellectual disability [1], results from a trinucleotide repeat expansion in the 5′-untranslated region of the *Fragile X Mental Retardation 1 (FMR1)* gene [2] which causes the transcriptional silencing and loss of its encoded protein, the fragile X mental retardation protein (FMRP) [3,4]. As a result, there is an uncontrolled activity of group I metabotropic glutamate receptors (mGluR) [5,6] and a reduced γ-aminobutyric acid-ergic (GABAergic) transmission [7,8], which seem to play a causal role in FXS deficits. The endocannabinoid system (ECS), a neuromodulatory system widely distributed in the brain, fine-tunes excitatory and inhibitory synaptic transmission by providing an exquisite level of fine local control over neurotransmitter release associated to synaptic contacts in specific brain areas [9]. Interestingly, several behavioral responses affected in FXS, such as cognition, neuronal excitability/plasticity, and nociception, depend on the activity of the ECS [9]. This system is composed of G-protein coupled receptors (mainly Cannabinoid type 1 (CB1), and Cannabinoid type 2 (CB2) receptors), fatty acid derived neurotransmitters (endocannabinoids, mainly anandamide (AEA) and 2-arachidonoylglycerol (2-AG)), and enzymes involved in the synthesis and degradation of these endocannabinoids, [9,10]. CB1 receptors are profusely expressed in presynaptic terminals in glutamatergic and more abundantly in GABAergic cells [9,11], where they preclude the release of neurotransmitters upon stimulation by endocannabinoids. Notably, postsynaptic activation of metabotropic glutamate receptor 5 (mGluR5) is a key physiological mechanism that promotes the synthesis of endocannabinoids in response to sustained synaptic activity [12], thereby triggering endocannabinoid/CB1 receptor-mediated long-term depression (eLTD) of excitatory and inhibitory transmissions [9]. Several studies have implicated alterations in glutamatergic and/or GABAergic neurotransmission in FXS [13,14,15], supporting the notion that loss of FMRP results in aberrant mGluR5 signaling pathways. In this regard, deregulated mGluR5-driven eLTD has been recently described in several brain areas of adult Fragile X Mental Retardation 1 (Fmr1) knockout (KO) mice [16,17]. These findings point to the possibility that defective endocannabinoid modulation of synaptic function may contribute to the phenotypic traits of FXS.

Rimonabant (5-(4-chlorophenyl)-1-(2,4-dichloro-phenyl)-4-methyl-*N*-(piperidin-1-yl)-1*H*-pyrazole-3-carboxamide, SR141716A), was the first selective CB1 receptor antagonist/inverse agonist [18] developed as an anti-obesity drug (Acomplia, Sanofi Aventis) on the premise that blocking cannabinoid activity reduces food intake [10]. After approval by the European Medicines Agency in 2006 as an anti-obesity treatment, it was withdrawn from the market two years later due to significant central effects, such as depression, anxiety, and suicidal ideation. The adverse effects of rimonabant occurred in 13%–16% of obese patients taking the effective dose of 20 mg/day, whereas their incidence was reduced in patients receiving 5 mg/day, a dose that showed reduced anti-obesity effects [19,20]. Importantly, rimonabant has inverse agonist properties on CB1 receptors at high doses [21], which has been postulated to underlie some of the side effects associated to this antagonist [22]. Therefore, other approaches to target CB1 receptors involve drugs with no inverse agonist properties such as NESS0327, a selective high affinity neutral antagonist [23].

The present study shows that rimonabant treatment normalizes anomalous synaptic plasticity in the hippocampus of Fmr1 KO mice at very low doses that could avoid previously reported side effects of this antagonist/inverse agonist. This improvement, together with the normalization of the object-recognition memory impairment in response to low intercalated doses of rimonabant and the similar efficacy of NESS0327, point to the beneficial effects of very low doses of CB1 receptor blockers independently of their inverse agonist activity.

## 2. Materials and Methods

### 2.1. Animals

Fmr1 knockout (KO) mice in Friend Virus B (FVB) background (Fmr1 KO, FVB.129P2-Pde6b+ Tyrc-ch Fmr1tm1Cgr/J) and wild-type mice (WT, FVB.129P2-Pde6b+ Tyrc-ch/AntJ) were purchased from The Jackson Laboratory (Bar Harbor, ME, USA) and crossed to obtain Fmr1 KO and WT littermates used in this study. All experimental animals were bred in-house at the Barcelona Biomedical Research Park (PRBB) Animal Facility. Fmr1 KO and WT male mice were used at 12–16 weeks of age. Mice were housed four per cage in a temperature (21 ± 1 °C) and humidity (55% ± 10%) controlled environment. Food and water were available ad libitum.

All the experiments were performed during the light phase of a 12 h light/dark cycle (lights on at 8 a.m. and off at 8 p.m.). Animals were handled for one week before starting the experiments. All animal procedures followed the standard ethical guidelines (European Communities Directive 86/60-EEC) and were approved by the local ethical committee (Comitè Ètic d'Experimentació Animal-Parc de Recerca Biomèdica de Barcelona, CEEA-PRBB). The PRBB has a standing Public Health Service approved Animal Welfare Assurance (no. A5388-01) granted by the Office of Laboratory Animal Welfare (OLAW) of the National Institutes of Health (USA). All behavioral tests were performed by observers blind to the different experimental groups.

### 2.2. Drugs

Rimonabant was obtained from Sanofi-Aventis (Sanofi-Aventis Recherche, Montpellier, France); NESS0327 was purchased from Santa Cruz Biotechnology, Santa Cruz, CA, USA). Both rimonabant and NESS0327 were dissolved in 5% ethanol: 5% cremophor-EL: 90% saline. Compounds were injected intraperitoneally (i.p.) at the doses indicated in a volume of 10 mL per kg.

### 2.3. Behavioral Tests

Object-recognition memory was assayed in the V-maze as described previously [24]. On day 1, mice were habituated to the empty maze for 9 min. On the second day, mice were introduced in the maze for 9 min, where two identical objects were presented. For the memory test, mice were placed again in the V-maze at the indicated time points for a period of 9 min, where one of the familiar objects was replaced by a novel object, and the total time spent exploring each of the two objects (novel and familiar). Object exploration was defined as the orientation of the nose towards the object at a distance of less than 2 cm. A discrimination index (DI) was calculated as the difference between the time spent exploring either the novel (Tn) or familiar (Tf) object divided by the total time exploring both objects (Tn + Tf) (DI = (Tn − Tf)/(Tn + Tf)). A discrimination index of 0 indicates no preference for any object and a DI higher than 0.3 was considered to reflect memory retention for the familiar object. Long-term object-recognition memory was assessed 24 h after the training session. Drug treatments were always performed after the training session to avoid possible intrinsic effects of the drugs during the acquisition phase.

### 2.4. Electrophysiology

Wild-type and Fmr1 KO mice aged 12–14 weeks were anesthetized with isoflurane and decapitated according to institutional regulations. Brains were removed to a chilled sucrose-based solution (in mM: 215 sucrose, 2.5 KCl, 26 NaHCO_3_, 1.6 NaH_2_PO_4_, 1 CaCl_2_, 4 MgCl_2_, 4 MgSO_4_, 20 glucose) and coronal brain slices (350 μm thick) were cut with a Vibratome Series 3000 Plus-Tissue Sectioning System (Ted Pella, Inc., Redding, CA, USA). Sections containing the hippocampus were allowed to recover by incubating them for 30 min at 32 °C in a solution containing the following (in mM: 62 NaCl, 2.5 KCl, 25 NaHCO_3_, 1.4 NaH_2_PO_4_, 1.1 CaCl_2_, 3.3 MgCl_2_, 2 MgSO_4_, 15 glucose, and 108 sucrose). Slices were then stored for at least 2 h at 32 °C in artificial cerebral spinal fluid (aCSF) (in mM: 124 NaCl, 2.5 KCl, 25 NaHCO_3_, 1.2 NaH_2_PO_4_, 2.5 CaCl_2_, 1.3 MgCl_2_ and 10 glucose). Experiments were conducted in a submersion-type recording chamber perfused at 2.5 mL/min with aCSF at 32 °C. All solutions were saturated with 95% O_2_ and 5% CO_2_, pH 7.4.

Field excitatory postsynaptic potentials (fEPSPs) were recorded from CA1 stratum radiatum using a borosilicate pipette filled with aCSF (1–2 MΩ). Synaptic responses were elicited by stimulation of the Schaffer collateral afferents with 150 μs duration pulses delivered through a bipolar platinum–iridium stimulation electrode (FHC, CE2C55). Input–output curves were constructed by plotting fEPSP slopes as a function of stimulation intensities ranging from 0 to 200 μA in increments of 20 μA. After the input–output study, baseline stimulation was delivered every 20 seconds at ~50%–60% of the maximum fEPSP for at least 20 min to ensure stability of the response. Group I mGluR-LTD was induced by bath application of (S)-3,5-Dihydroxyphenylglycine (DHPG) (Sigma-Aldrich Química S.L., Madrid, Spain) (100 µM, 5 min) and responses were recorded for 1 h after plasticity induction. Recordings were performed with a MultiClamp 700B (Axon Instruments, Sunnyvale, CA, USA), and output signals were filtered at 3 KHz. Data were digitized (10 KHz) on a DigiData 1332A (Axon Instruments) and collected using Clampex 9.2 (Axon Instruments, Sunnyvale, CA, USA). To measure the stability of synaptic responses in each experiment the initial slope of the fEPSP was expressed as a percentage of baseline average. Maximal transient depression (MTD) was calculated at the time point post-DHPG application with the greatest depression, and LTD was determined by averaging responses recorded 55–60 min after addition of DHPG. Two to three slices were tested from a given mouse, and no fewer than three mice were used in any experimental group.

### 2.5. Statistical Analysis

Results are reported as mean ± standard error of the mean (SEM). Statistical comparisons of behavioral data were performed using the Statistica Software (StatSoft). Dose-dependence experiments in Fmr1 KO mice were evaluated by one-way analysis of variance (ANOVA). The rest of the behavioral studies were evaluated using two-way ANOVA (treatment and genotype as factors) followed by the Newman–Keul’s post-hoc test. Statistical comparisons of electrophysiological data were performed using unpaired Student’s *t* test with GraphPad Prism software (GraphPad Software). Each slice was considered as an individual *n*. Comparisons were considered statistically significant when *p* < 0.05.

## 3. Results

### 3.1. Low Doses of Rimonabant Ameliorate the Cognitive Impairment in Fmr1 KO Mice

**Figure 1:**
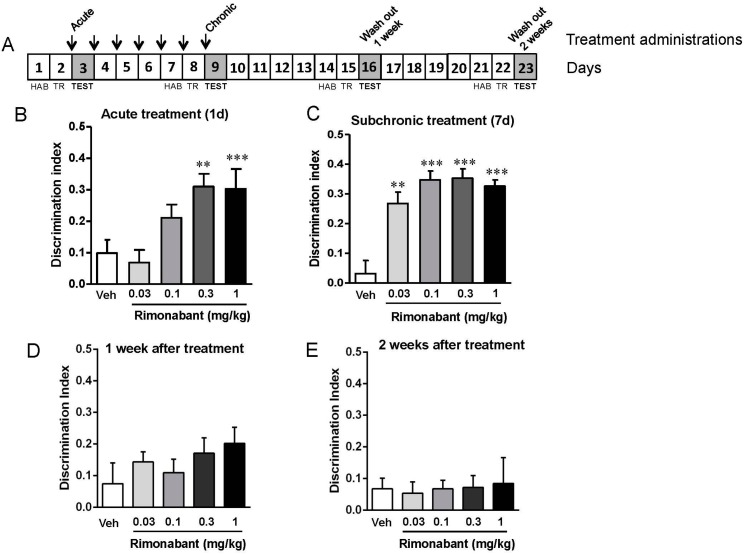
Low doses of rimonabant ameliorate the cognitive impairment present in the Fragile X Mental Retardation 1 (Fmr1) knockout (KO) mice. (**A**) Fmr1 KO mice were tested in the novel object recognition memory test after 1 day (Veh, *n* = 4; 0.03 mg/kg, *n* = 7; 0.1 mg/kg, *n* = 6; 0.3 mg/kg, *n* = 7; 1 mg/kg, *n* = 5); (**B**) and 7 days (Veh, *n* = 4; 0.03 mg/kg, *n* = 7; 0.1 mg/kg, *n* = 6; 0.3 mg/kg, *n* = 7; 1 mg/kg, *n* = 5); (**C**) of treatment with different doses of rimonabant (0.03 mg/kg, 0.1 mg/kg, 0.3 mg/kg, 1 mg/kg) or its vehicle. Drugs were administered after the training phase and discrimination indexes were obtained 24 h after training. Mice were also tested in the object recognition memory test 1 week (Veh, *n* = 4; 0.03 mg/kg, *n* = 7; 0.1 mg/kg, *n* = 7; 0.3 mg/kg, *n* = 7; 1 mg/kg, *n* = 5); (**D**) and 2 weeks (Veh, *n* = 4; 0.03 mg/kg, *n* = 7; 0.1 mg/kg, *n* = 6; 0.3 mg/kg, *n* = 7; 1 mg/kg, *n* = 5); (**E**) after the end of the treatment. Abbreviations: Veh, vehicle; HAB, habituation; TR, training. Data are expressed as mean ± SEM. ** *p* < 0.01 and *** *p* < 0.001 (rimonabant vs. vehicle treatment).

Fmr1 KO mice were analyzed in the object-recognition memory test after a single administration of rimonabant at different doses (0.03, 0.1, 0.3, and 1 mg/kg) compared to vehicle (Figure 1A,B). Interestingly, a single administration of rimonabant produced significant improvements at 0.3 mg/kg (Student’s *t*-test, *t* = −3.358, *p* < 0.01, *df* = 9) and 1 mg/kg (Student’s *t*-test, *t* = −5.748, *p* < 0.001, *df* = 7). Doses lower than 0.3 mg/kg also showed a significant effect (0.03 mg/kg: Student’s *t*-test, *t* = −3.891, *p* < 0.01, *df* = 9; 0.1 mg/kg: Student’s *t*-test, *t* = −6,219, *p* < 0.001, *df* = 8) compared to vehicle control after 7 days of chronic treatment (Figure 1A,C). Notably, repeated treatment with rimonabant did not affect overall exploration time in the object-recognition test, but improved discrimination index in the Fmr1 KO mice compared to vehicle-treated controls. In these same mice, object recognition memory was assessed 7 and 14 days later, under conditions where mice did not receive any treatment (Figure 1A,D–E). We found that this washout period reduced the previous beneficial effect of rimonabant treatment when object-recognition memory was analyzed at 7 days (Figure 1D) and 14 days (Figure 1E) after rimonabant treatment was stopped. Under these circumstances, none of the previous treatment conditions produced remaining effects, showing that rimonabant administration must be maintained to attain cognitive improvement.

### 3.2. Rimonabant at a Low Dose Normalizes LTD in Fmr1 KO Mice

**Figure 2:**
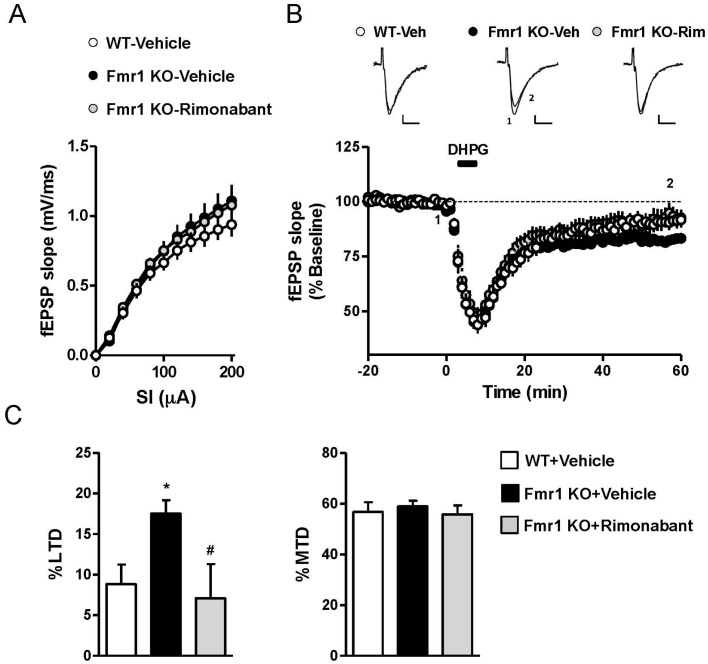
Rimonabant rescues enhanced long-term depression (LTD) in Fragile X Mental Retardation 1 (Fmr1) knockout (KO) mice. Input–output function (**A**) and group I metabotropic glutamate receptor (mGluR) LTD; (**B**,**C**) were evaluated in hippocampal slices from mice treated with rimonabant (0.1 mg/kg) or its vehicle for 7 days. (**A**) Plots of field excitatory postsynaptic potentials (fEPSPs) elicited in the CA1 region by stimulation of Schaffer collateral projections were not detectably different between Fmr1 KO and wild-type (WT) mice. Subchronic treatment with rimonabant did not modulate basal field responses in Fmr1 KO mice; (**B**) Time-course of fEPSP slopes following bath application of the group 1 mGluR agonist (S)-3,5-Dihydroxyphenylglycine (DHPG) (100 µM, 5 min). (**C**) LTD and maximum transient depression (MTD) calculated from the experiments depicted in (**B**). LTD was enhanced in Fmr1 KO mice (* *p* < 0.05 vs. WT mice) and was rescued by subchronic rimonabant (^#^ *p* < 0.05 vs. vehicle treatment). Data are expressed as mean ± SEM of 6–10 slices from 3 to 5 mice per condition. Scale bars: 0.1 mV/10 msec.

Elevated group I mGluR-LTD is considered a key phenotype of Fmr1 KO mice based on the observation that genetic and pharmacological manipulations that attenuate behavioral and cognitive abnormalities often normalize this form of synaptic plasticity [6,25,26]. Here we tested whether in vivo administration of rimonabant could reduce the elevated ex vivo group I mGluR-LTD in the Fmr1 KO mice hippocampus. WT and Fmr1 KO animals received a daily dose of rimonabant (0.1 mg/kg, i.p.) or vehicle for 7 days until the day before euthanasia and hippocampal slice preparation. Basal synaptic transmission was not detectably different between vehicle-treated WT and Fmr1 KO mice, as assessed by input–output curves for fEPSP elicited in the CA1 region by stimulation of Schaffer collaterals (NS, repeated measures ANOVA) (Figure 2A). Chronic administration of rimonabant did not alter the efficacy of basal synaptic transmission in Fmr1 KO mice (NS, repeated measures ANOVA) (Figure 2A). Bath application of DHPG (100 µM, 5 min) induced a transient acute depression, which represents an electrophysiological readout of group I mGluR activation, followed by a small LTD in WT mice treated with vehicle (91.2% ± 2.4% baseline 55–60 min after DHPG application) (Figure 2B,C). Consistent with previous reports, LTD induced by DHPG was significantly enhanced in the vehicle-treated KO animals (82.5% ± 1.7%; % baseline; *p* < 0.05, unpaired *t* test versus wild-type mice), whereas the maximum transient depression (MTD) in response to the group I mGluR agonist was similar between wild-type and mutant mice (Figure 2B,C). Treatment with rimonabant did not modulate LTD by DHPG, but normalized the elevated LTD induced by the group I mGluR agonist in slices from Fmr1 KO mice (92.9% ± 4.2%; *p* < 0.05, unpaired *t* test versus vehicle treated KO mice) (Figure 2B,C).

### 3.3. Discontinuous Treatment with a Low Dose of Rimonabant Reaches Normalization of the Cognitive Performance

**Figure 3:**
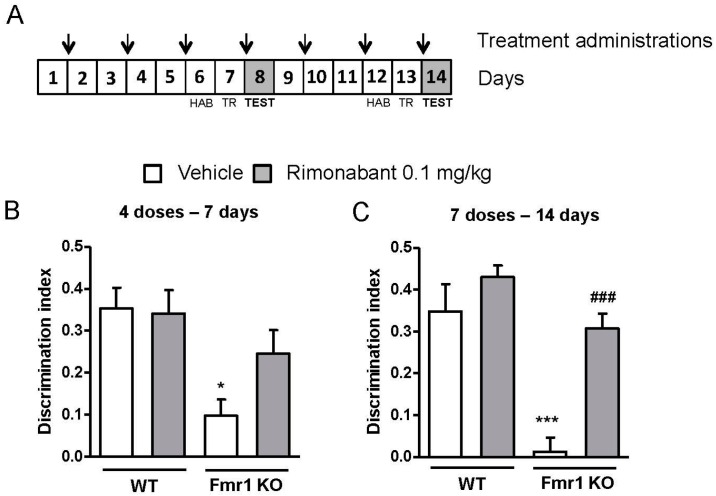
Discontinuous treatment with a low dose of rimonabant normalizes the cognitive impairment observed in the Fmr1 KO mice. (**A**) Mice were treated with a low dose (0.1 mg/kg) of rimonabant or its vehicle every two days during 14 days. Mice were exposed to the novel object recognition test after (**B**) 4 administrations (7 days) (WT-Veh, *n* = 4; WT-Rim, *n* = 6; Fmr1KO-Veh, *n* = 3; Fmr1KO-Rim, *n* = 5) and (**C**) after 7 administrations (14 days) (WT-Veh, *n* = 4; WT-Rim, *n* = 6; Fmr1KO-Veh, *n* = 3; Fmr1KO-Rim, *n* = 5) of vehicle or rimonabant. On day 7 and 14, drugs were administered after the training phase and discrimination indexes were obtained 24 h after training. Abbreviations: Veh, vehicle; Rim, rimonabant; HAB, habituation; TR, training. Data are expressed as mean ± SEM. * *p* < 0.05 and *** *p* < 0.001 (Fmr1 KO vs. WT mice); ^###^ *p* < 0.001 (rimonabant vs. vehicle treatment).

In order to assess whether rimonabant doses could be further reduced, we used a schedule where drug administration was performed every other day. Fmr1 KO and wild-type mice received rimonabant at the dose of 0.1 mg/kg every 2 days during 14 days (7 administrations, Figure 3A). Mice were tested for object-recognition memory after 7 (4 administrations, Figure 3A,B) and 14 days (7 administrations, Figure 3A,C). Memory assessment did not reveal any significant improvement after 4 rimonabant administrations (genotype: F(1,14) = 9.407, *p* = 0.008; treatment: F(1,14) = 1.414, *p* = 0.254; interaction: F(1,14) = 1.946, *p* = 0.184) (Figure 3B) in the Fmr1 KO group. Interestingly, 7 administrations in alternating days significantly improved the performance of Fmr1 KO mice compared to vehicle treated mice (genotype: F(1,14) = 32.633, *p* < 0.001; treatment: F(1,14) = 22.49, *p* < 0.001; interaction: F(1,14) = 7.88, *p* = 0.013). This set of data reveals the possibility to reduce the dose of rimonabant up to 20 times below its use described in our previous study [24] with satisfactory results in cognitive performance.

### 3.4. The CB1 Receptor Neutral Antagonist NESS0327 Also Has Beneficial Effects in Object-Recognition Memory

**Figure 4:**
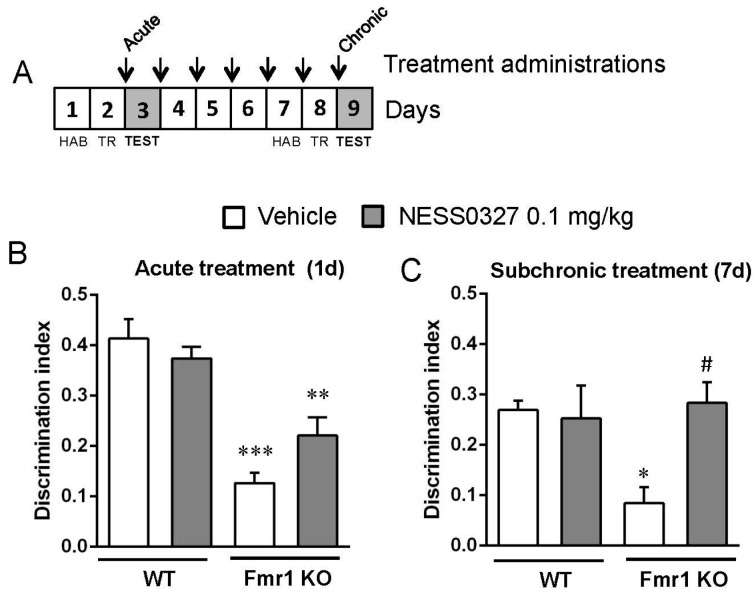
The cannabinoid type 1 (CB1) receptor neutral antagonist NESS0327 also ameliorates the cognitive deficit observed in the Fmr1 KO mice. (**A**) Fmr1 KO mice were tested in the novel object recognition memory test after acute (WT-Veh, *n* = 7; WT-NESS, *n* = 7; Fmr1KO-Veh, *n* = 4; Fmr1KO-NESS, *n* = 7) (**B**) and subchronic (**C**) treatment with NESS0327 (0.1 mg/kg, i.p.) or its vehicle. Drugs were administered after the training phase and discrimination indexes were obtained 24 h after training. Abbreviations: HAB, habituation; TR, training. Data are expressed as mean ± SEM. ** *p* < 0.01, *** *p* < 0.001 (Fmr1 KO vs. WT mice); ^##^ *p* < 0.01 (NESS0327 vs. vehicle treatment).

We assessed whether the CB1 receptor neutral antagonist, NESS0327, would modify the cognitive deficit observed in the Fmr1 KO mice (Figure 4A), in a similar manner to rimonabant, in order to find out if the inverse agonist characteristic of rimonabant over CB1 receptors would be relevant for targeting the cognitive deficit in the fragile X syndrome mouse model.

A single administration of NESS0327 (0.1 mg/kg) did not significantly modify the performance of Fmr1 KO mice compared to those treated with vehicle (genotype: F(1,21) = 41.91, *p* < 0.001; treatment: F(1,21) = 0.65, *p* = 0.429; interaction: F(1,21) = 3.915, *p* = 0.061) (Figure 4B). Instead, the repeated administration of NESS0327 (0.1 mg/kg) for 7 days normalized the object-recognition memory performance compared to wild-type controls (genotype: F(1,20) = 2.461, *p* = 0.132; treatment: F(1,20) = 3.446, *p* = 0.07; interaction: F(1,20) = 4.837, *p* = 0.039) (Figure 4C). These data further support the potential use of molecules targeting the CB1 receptor and avoiding the inverse agonist properties of rimonabant that could be linked to the negative side effects.

## 4. Discussion

In the present study, we describe that lower doses than those previously reported [24] of the CB1 receptor antagonist/inverse agonist rimonabant, or a low dose of the CB1 receptor neutral antagonist NESS0327 (0.1 mg/kg), prevent the cognitive deficit in the Fmr1 KO mice measured in the novel object-recognition memory test. This beneficial effect of rimonabant correlated with the normalization of mGluR-LTD in hippocampal slices from Fmr1 KO mice.

Rimonabant was the first compound described with high affinity and specificity for CB1 receptors, showing an antagonist/inverse agonist profile [18]. It was developed as a treatment for obesity, but after reaching the market in 2007 it was withdrawn two years later due to its psychiatric side effects [19]. The doses of rimonabant studied in clinical trials, 5 and 20 mg/day, demonstrated dose-dependent adverse effects. Taking into account the dose conversion between species proposed by Reagan-Shaw et al. in 2007 [27], the dose of 5 mg/day for a 70 kg person would correspond to 0.88 mg/kg in mice, whereas 20 mg/day corresponds to 3.5 mg/kg. Such doses would be respectively 8.8 and 35 times higher than the dose used in this study to normalize novel object-recognition memory and hippocampal synaptic plasticity (0.1 mg/kg). Our study also reveals that rimonabant doses can be reduced further by administering the drug every two days to obtain the same amelioration on the memory deficit present in the Fmr1 KO mice, and an even lower dose of rimonabant (0.03 mg/kg) also improved the cognitive deficit. These low doses of rimonabant in mice are far from those showing food intake reduction (1 mg/kg) [28]. Altogether, our data suggests the possibility of using a dose of rimonabant at least 35 times lower than the dose affecting mood in humans treated with this drug for obesity. In contrast to these side effects, such a low dose of rimonabant (0.1 mg/kg) has been found to have anxiolytic properties in mice [29].

Rimonabant shows mixed antagonist and inverse agonist properties [30]. The adverse effects of rimonabant on anxiety have been proposed to derive from its inverse agonist properties, since higher doses of rimonabant than those used in the present study increased anxiety, an effect not observed by the use of the neutral antagonist NESS0327 [31]. It is worth mentioning that drugs with an inverse agonist profile over CB1 receptors have been shown to increase GABAergic neurotransmission in hippocampal slices [32], which would be related to the potential of inverse agonist to suppress constitutive activity of CB1 receptors [31]. Instead, NESS0327 did not affect GABAergic release [32] and did not suppress constitutive activity of CB1 receptors under similar conditions [31]. These differences in the pharmacodynamic profile do not seem to be relevant for improving the cognitive deficit in the Fmr1 KO mouse model, since both rimonabant and NESS0327 showed similar beneficial effects.

Group I mGluR-LTD is a major form of synaptic plasticity underlying learning and memory processes. In the CA1 region of the hippocampus group I mGluR-LTD can be pharmacologically triggered by DHPG [33] and its expression is mediated by a reduction in surface α-amino-3-hydroxy-5-methyl-4-isoxazolepropionic acid (AMPA) receptors following the activation of a variety of signaling pathways [34]. Enhanced hippocampal group I mGluR-LTD is a well-described feature of Fmr1 KO mice [35], which eventually led to the hypothesis that such anomalous synaptic plasticity could be related to the intellectual disability in FXS [5], and that treatments targeting mGluR5 could improve the cognitive deficit in FXS [36]. Although preclinical studies following this hypothesis were significantly successful [6], clinical trials exploring the possibility to use mGluR5 as a target for FXS treatment have not been efficacious to date. It is worth mentioning, though, that normalization of group I mGluR-LTD is a hallmark of several pharmacological and genetic manipulations that correct behavioral abnormalities in Fmr1 KO mice [25,37,38]. In the present study, we have used rimonabant at a dose close to 30–100 times lower (0.1 mg/kg, subchronic) than that used in experimental animals to reduce body weight (3–10 mg/kg, chronic) [39]. This low dose of rimonabant remediates synaptic plasticity alterations in the Fmr1 KO, which correlates with the normalization of the memory impairment in the object-recognition memory test.

In our study, rimonabant treatment was stopped the day before mice were used for recordings, indicating that its effect may be related with long lasting alterations in brain circuits. This is reminiscent of the results obtained in the normalization of DHPG-LTD in the hippocampus, with other approaches such as 2-chloro-4-((2,5-dimethyl-1-(4-(trifluoromethoxy)phenyl)-1H-imidazol-4-yl)ethynyl) pyridine (CTEP) [6] that normalized specific features in Fmr1 KO mice. Other potential treatments to be assayed in FXS have been first tested for synaptic plasticity normalization, such as the agonist for serotonin 5-HT7 receptors [40], on the bases that enhanced mGluR5-LTD is a synaptic plasticity landmark in FXS.

It cannot be discarded that other approaches targeting the CB1 receptors beyond those assessed in the present study may also show beneficial effects in FXS models. In this regard, other compounds acting on CB1 receptors, such as allosteric modulators [41] or even specific components of the *Cannabis sativa* plant such as cannabidiol, that may act as CB1 receptor negative modulators [42] are alternative approaches worth exploring. Similarly, future studies on CB1 receptor targeting should address the possibility of starting the pharmacological intervention earlier during neurodevelopment to further improve the therapeutic potential of such an intervention. Other important steps ahead are the use of other animal models with improved translational value, such as the rat model of FXS [43], since results in mice have not managed to directly predict effectiveness in the clinical setting.

Altogether our results indicate that the benefits of CB1 receptor blockade in the cognitive performance of Fmr1 KO mice can be obtained with doses of rimonabant much lower than those reported to cause potential adverse effects. These beneficial effects of rimonabant are associated to its ability to normalize anomalous synaptic plasticity.
